# Cerebrospinal Fluid Biomarkers in Parkinson’s Disease: A Critical Overview of the Literature and Meta-Analyses

**DOI:** 10.3390/brainsci10070466

**Published:** 2020-07-20

**Authors:** Takayuki Katayama, Jun Sawada, Kae Takahashi, Osamu Yahara

**Affiliations:** 1 Department of Neurology, Asahikawa City Hospital, 1-1-65 Kinseicho, Asahikawa 070-8610, Japan; kae.t-452@live.jp (K.T.); oyahara@kca.biglobe.ne.jp (O.Y.); 2 Department of Neurology, Asahikawa Medical University Hospital, Asahikawa 078-8510, Japan; sawajun@asahikawa-med.ac.jp

**Keywords:** Parkinson’s disease, cerebrospinal fluid, α-synuclein, amyloid β42, tau, neurofilament light chain

## Abstract

Parkinson’s disease (PD) is a common neurodegenerative disorder; however, well-established biochemical markers have not yet been identified. This review article covers several candidate cerebrospinal fluid (CSF) biomarkers for PD based on the recent literature and meta-analysis data. The decrease of α-synuclein in PD is supported by meta-analyses with modest reproducibility, and a decrease of amyloid β42 is seen as a prognostic marker for cognitive decline. Tau, phosphorylated tau (p-tau), and neurofilament light chains have been used to discriminate PD from other neurodegenerative disorders. This article also describes more hopeful biochemical markers, such as neurotransmitters, oxidative stress markers, and other candidate biomarkers.

## 1. Introduction

Parkinson’s disease (PD) is one of the most common neurodegenerative disorders, and is characterized by resting tremors, bradykinesia, rigidity, and postural instability, as well as cognitive symptoms [1]. Pathologically, this disorder is defined by degeneration of the substantia nigra and the presence of Lewy bodies, which contain abundant amounts of α-synuclein. Researchers have also found that gene mutations of α-synuclein cause familial forms of PD. Therefore, α-synuclein is considered to play a major role in the pathogenesis of PD.

The current clinical diagnostic criteria of PD are based on existence of parkinsonism and the exclusion of other disorders, and all the tests for PD, such as magnetic resonance imaging or nuclear medicine imaging are positioned as supportive tools [2]. Therefore, good biomarkers for PD with high sensitivity and specificity are desired.

The cerebrospinal fluid (CSF) biomarkers for PD have been investigated widely to elucidate the pathophysiology and to support differentiating PD from normal subjects or other neurological disorders, such as multiple system atrophy (MSA) [3] or progressive supranuclear palsy (PSP) [4]. CSF biomarkers are also sought for the evaluation of disease activity/progression, severity, staging, comorbidity (i.e., cognitive dysfunction), and prognosis.

Confirmed CSF biomarkers have not yet been established; however, some candidate molecules (for example, α-synuclein) have been inspected as potential ones. In this article, we aimed to provide a comprehensive overview for the biomarkers for PD and referred to recent meta-analyses for several biomarkers.

## 2. Overview and Classification of the CSF Biomarkers of PD

A previous review divided the biomarker candidates into six categories (A, neurotransmitters and neuromodulators; B, oxidative stress markers; C, inflammatory and immunological markers; D, growth factors; E, proteins involved in PD pathology; and F, others) [5]. Under each category, the authors focused on their usability in these four clinical domains (1, distinguishing PD from controls; 2, distinguishing PD from other neurodegenerative diseases; 3, representing the disease severity or cognitive abilities; and 4, being of prognostic value regarding the disease severity and cognitive abilities).

Our review also adopted this categorization; however, the clinical usability was divided into three clinical domains (1, 2, and 3 + 4). This categorization is tentative and conceptual, and exceptions are possible (e.g., one marker can belong to two categories, or be not categorical). We also annotated the putative functions, roles, or pathophysiological interpretations for each marker in Table 1 of this article to provide perspectives on this field. Any single marker cannot cover all these domains as yet. This is unsurprising as the central nervous system in PD is exposed to various pathological changes (aging, synuclein-, amyloid-, tau-pathology, etc.). Therefore, many recent studies have adopted combinations of several CSF biomarkers.

## 3. Materials and Methods

This study is based on a literature search in the database PubMed with the keywords (Parkinson AND cerebrospinal fluid AND biomarker AND (review OR meta-analysis)) on 24 May 2020. Non-English publications were excluded from this review because of the difficulty of assessing the contents as available open resources. Only the most relevant or earliest studies were included in this review and duplicated contents were omitted as well as studies with a small sample size (n < 20).

**Figure 1:**
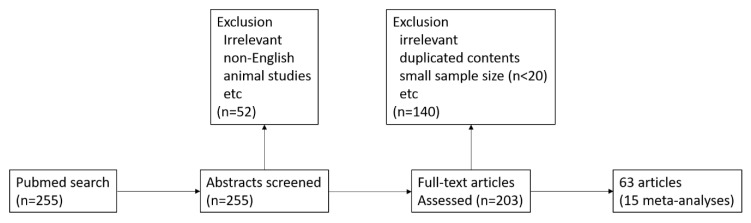
Flowchart of the article selection process.

Of the 255 articles identified in the database, 63 were included in this study (Figure 1). The search also found 15 meta-analysis articles referring to α-synuclein, neurofilament light chains, cytokines (interleukin (IL)-1β, IL-6, and transforming growth factor (TGF)-β1), amyloid Aβ42, tau, and phosphorylated tau.

We also focused on the following markers in particular: (1) α-synuclein and its related molecules; (2) dopamine, neurotransmitters, and the metabolites; (3) oxidative stress markers; (4) amyloid beta as a predictor for cognition; and (5) tau, phosphorylated tau, and neurofilament light chains as discriminators for atypical Parkinsonism.

## 4. Results

The results are summarized in Table 1 (the biomarkers for distinguishing PD from controls), Table 2 (the biomarkers for distinguishing PD from other neurodegenerative disorders), and Table 3 (the biomarkers for representing disease severity or cognitive abilities). Then, we made detailed comments for each marker category.

### 4.1. Neurotransmitters and Neuromodulators: Focusing on Dopamine and the Metabolites

The depletion of dopaminergic neurons in the substantia nigra is an essential pathology in PD, and noradrenergic neurons in locus ceruleus were also observed. Dopamine and noradrenaline decreases are also potential candidates of PD, as well as the metabolites, including dihydroxyphenyl acetic acid (DOPAC) and homovanillic acid (HVA). However, the levels of these compounds actually have different sources and meanings [7]. CSF homovanillic acid is rather distantly related to neuronal dopamine stores and reflects several intervening processes. As dopaminergic neurons do not contain catechol-O-methyltransferase, CSF homovanillic acid depends on the uptake and intracellular O-methylation in extra-dopaminergic cells. Thus, in PD, the striatal content of homovanillic acid is not as severely decreased as that of dopamine. CSF dopamine also may not provide an accurate reflection of central dopamine deficiency. Dopamine in extracellular fluid is derived mainly from exocytotic release in response to pathway traffic and the escape of neuronal reuptake by the cell membrane dopamine transporter.

As dopaminergic neurons are lost, pathway traffic to the remaining terminals likely increases compensatorily, thereby augmenting dopamine delivery from those terminals to the extracellular fluid; thus, CSF dopamine may underestimate the extent of the loss of neuronal dopamine stores. CSF dopamine concentrations are infinitesimal—sometimes below the detection limit of the assay method [7]. Meta-analysis articles were not found in the PubMed database in this category. CSF catecholamine would be influenced by levodopa intake, and, therefore, the measurements should be performed for a levodopa-naïve or washed-out state.

Other marker changes (anandamide, neuromodulin (GAP43), 3-hydroxykynurenine, orexin, and 5-hydroxyindole acetic acid (5-HIAA)) were also reported [6,8,9,45,54].

### 4.2. Oxidative Stress Markers

Numerous markers have been investigated to evaluate oxidative stress in PD [10,11,12,13,14,15,16,17,18,19,20,21,22,23,24,55] (Table 1 and Table 2); however, meta-analysis showed that only five CSF oxidative stress markers, 8-hydroxy-2’-deoxyguanosine (8-OHdG), Mn, Cu, Zn, and Fe, could be evaluated, and no significant difference was found between PD patients and controls [13]. On the other hand, the meta-analysis showed that patients with PD had significantly higher levels of blood oxidative stress markers compared with healthy control subjects for ferritin, 8-OHdG, nitrite, and malondialdehyde; meanwhile, the concentrations of uric acid, catalase, glutathione, and total-cholesterol were significantly lower in PD patients compared with healthy control subjects [13]. Oxidative stress markers could be influenced by numerous confounding factors, such as age, sex, diets, drugs, supplements, smoking, exercise, and comorbidities (hypertension, diabetes mellitus, inflammation, ischemia, etc.).

Other markers (advanced oxidized protein products (self-oxidized), ceruloplasmin ferroxidase activity, oxidized Q10, Cu/Zn-superoxide dismutase, DJ-1, glutathione S-transferase Pi, glutathione (oxidized), hydroxy radical (^·^OH), lipid peroxidation, nitrites, nitrates, silicic acid, xanthine, 3-nitrotyrosine products, and 8-hydroxyguanosine (8-OHG)) have also been investigated [10,11,12,13,14,15,16,17,18,19,20,21,22,23,24,55].

### 4.3. Inflammatory and Immunological Markers

Researchers have suggested that cytokine-mediated inflammation plays a key role in the onset and/or development of PD. One meta-analysis reported that IL-1β, IL-6, and TGF-β were elevated in PD [27] and the work also revealed the unique inflammatory response profile in the central nervous system of patients with Alzheimer’s disease, PD, and amyotrophic lateral sclerosis. Other markers (β2-microglobulin, Immunoglobulin G (IgG) ratio (CSF/serum), interferon (IFN)-γ, prostaglandin E2, soluble CD (cluster of differentiation) 14, tumor necrosis factor (TNF)-α, and differentially sialylated isoforms of Serpin A1 and Flt3 (Fms-related tyrosine kinase 3) ligand) have also been investigated [6,19,25,26,28,29,56].

### 4.4. Growth Factors

Only sparse data was available for this area. Brain-derived neurotrophic factor (BDNF) was significantly different (decreased) between the control and both neurodegenerative groups (PD and Alzheimer’s disease) but not between neurodegenerative groups [25]. CSF progranulin was investigated in PD, amyotrophic lateral sclerosis and controls, but no difference was found among the groups [30].

### 4.5. Proteins Involved in PD Pathology

#### 4.5.1. α-Synuclein and Its Related Molecules

α-synuclein is considered one of the most important targets in this field, and many studies have investigated CSF α-synuclein. The reported control/normal CSF α-synuclein values varied widely; however, meta-analyses showed that the CSF levels of α-synuclein decreased in patients with PD [31,32,33,34]. One possible reason for the conflicting results is the contamination of CSF by α-synuclein from the blood, as α-synuclein is abundant in whole blood, plasma, and serum, in which its levels are up to 10^2^–10^3^ times higher than those found in the CSF [64]. Synuclein oligomer and phosphorylated α-synuclein are also candidate molecules supported by a meta-analysis [34]. The meta-analyses showed that the sensitivity and specificity of α-synuclein were 0.72–0.88% and 0.40–0.65, respectively [32,34]. The sensitivity and specificity of α-synuclein oligomer were 0.71 and 0.64, respectively [34] (Table 4).

A recent study demonstrated that CSF α-synuclein decreased early in the disease, preceding motor PD. CSF α-synuclein does not correlate with the progression and therefore does not reflect ongoing dopaminergic neurodegeneration. Decreased CSF α-synuclein may be an indirect index of changes in the balance between α-synuclein secretion, solubility, or aggregation in the brain, reflecting its overall turnover [65]. This corresponds with another finding that the Hoehn–Yahr stage was not correlated with the CSF α-synuclein level, and that the striatal binding ratio on dopamine transporter imaging with ^123^I-ioflupane decreased in the PD group, but this was not correlated with the CSF α-synuclein level [66]. Another area of interest regarding CSF α-synuclein is its relationship with cognitive function; however, the research remains inconclusive or less influential [66].

Technical caution and pitfalls for the measurement of α-synuclein should be considered for appropriate use. Perianalytical considerations for biomarker studies in PD have been summarized [67]. α-synuclein is abundant in erythrocytes and, therefore contained in the serum, plasma, and whole blood, and, thus, blood contamination should be avoided cautiously. CSF hemoglobin is often referred to as a marker for blood contamination. The use of traumatic needles, discarding the first five drops of CSF, and collection with polypropylene or siliconized tubes are recommended for sampling for CSF. There does not appear to be any diurnal fluctuation.

Why α-synuclein decreases in the CSF of PD remains unknown. Low production, aggregation in the brain, and/or increased clearance of α-synuclein would be possible.

#### 4.5.2. Amyloid Beta, Tau, and Phosphorylated Tau as Predictors for Cognition

Cognitive decline in PD is well known, and several studies reported a relationship between amyloid-beta and cognition. Another recent topic of research interest is the relationship between PD, amyloid β42 (Aβ42), tau, and p-tau, particularly from the viewpoint of cognitive function. One meta-analysis showed that the CSF Aβ42 level in PD with the cognitive impairment (CI) cohort was lower than that in PD with the normal cognition (NC) cohort. Reduced Aβ42 as well as elevated t-tau and p-tau were observed in the PD with dementia (PDD) cohort compared with the PDNC cohort. Therefore, the meta-analysis concluded that amyloid pathology and tauopathy may participate in the development of PDD, which is similar to Alzheimer’s disease [35].

It is unknown whether α-synuclein and amyloid beta can interact; however, a previous study reported no significant correlation between α-synuclein and amyloid beta [66]. There is no robust evidence regarding the contribution of α-synuclein to cognitive function. These matters should be investigated in further research.

#### 4.5.3. Neurofilament Light Chains for Discriminating PD from Other Neurological Disorders

Neurofilament light chains (NFL) also have the potential to differentiate PD from MSA or PSP, and this is supported by meta-analyses [57,58,59,60]. NFL elevation was observed in MSA, PSP, and corticobasal degeneration. In terms of the comparison between PD and atypical parkinsonian disorders, the sensitivity and specificity of NFL were 0.82 and 0.85, respectively [59] (Table 4).

#### 4.5.4. Other Proteins

Other proteins (neurosin, glial fibrillary acidic protein (GFAP), clusterin, neurofilament heavy chains, YKL-40 (CHI3L1), soluble neuron-glial antigen 2 (NG2) proteoglycan, ubiquitin, ubiquitin carboxyl-terminal hydrolase isozyme L1 (UCHL-1), apolipoprotein A1, apolipoprotein A2, apolipoprotein ε, transthyretin, glycan isoforms of transferrin (serum-type/brain-type ratio), neuron-specific enolase, myelin basic protein, heart fatty acid binding protein, glucocerebrosidase activity, and neprilysin activity) have also been investigated [18,25,28,36,37,38,39,40,41,42,43,44,46,62,63].

##### Others

Many other candidate markers have been reported (Table 1) [6,9,18,25,47,48,49,50,51,52,53,61]; however, these candidates also include preliminary ones with small sample data. These markers require verification and interpretation of the background. Pathophysiological meanings have been proposed for each marker, but these should also be inspected.

Recent studies focused on the hypothesis that epigenetic mechanisms contribute to PD development and progression. Epigenetics refers to the regulatory mechanisms of gene expression that are not mediated by the DNA sequence itself, but by chemical or allosteric DNA modifications or by the action of regulatory noncoding RNAs. MicroRNAs (miRNAs) are small noncoding RNAs that serve as posttranscriptional regulators of gene expression. They bind to messenger RNA (mRNA) and promote mRNA degradation and/or decrease the translation. One meta-analysis identified four studies for miRNA of CSF; however, no significant signals were found in the CSF, although the study identified 13 significantly differentially expressed miRNAs in the brain (n = 3) and blood (n = 10) [51]. The most compelling finding was miRNA hsa-miR-132-3p binding to the mRNA of the α-synuclein gene (SNCA).

## 5. Discussion

Cumulative data, including meta-analyses, support that a decrease of CSF α-synuclein is observed in PD and that a decrease of amyloid β42 is a predictor of cognitive decline in PD. Tau, phosphorylated tau, and neurofilament light chains were able to discriminate PD from other neurodegenerative disorders. The elevation of cytokines (IL-1β, IL-6, and TGF-β) in PD suggested pro-inflammatory mechanisms in the pathogenesis of PD. CSF DOPAC appeared to provide a sensitive means to identify PD. This review could not identify reliable CSF oxidative stress markers in the literature thus far.

Further studies are necessary to reveal α-synuclein abnormalities, which may include the transcription, translation, epigenetic regulation, and kinetics (production, distribution, post-translational modification, oligomerization, aggregation, clearance, etc.). The standardization of measurement techniques is also required to use the practical application of these markers.

This review has some limitations. First, the abovementioned meta-analyses showed statistical significance; however, heterogeneity was present in each meta-analysis. Overlapping of the data between PD and the counterpart is present in any marker; therefore, they should be applied carefully. Second, these CSF biomarker samplings were performed mainly while the patients were alive, and, therefore, the diagnosis at the time of CSF sampling was established with clinical criteria but not as a histopathological diagnosis.

It is inevitable to have the limitation of pre-mortem differential diagnosis of AD and dementia with Lewy bodies (DLB). Certain studies adopted post-mortem CSF sampling with neuropathologically diagnosed PD subjects [18]; however, post-mortem changes may happen in the CSF or even in the brain, and the post-mortem data should be compared with longitudinal information. Certain patients showed a mixed pathology of AD and Lewy pathology [68]. The employment of other surrogate markers, such as amyloid positron emission tomography (PET), tau PET, or dopamine transporter imaging, would be necessary for the verification of the clinical diagnosis, as well as other modalities, such as myocardium metaiodobenzylguanidine (MIBG) and dopamine transporter (DAT) scintigraphy. Longitudinal data sampling is also needed but limited [65].

## 6. Conclusions

In conclusion, the meta-analyses for cerebrospinal fluid biomarkers of PD showed: (1) a decrease of α-synuclein was a marker of PD, (2) a decrease of amyloid β42 was a marker of cognitive decline in PD, (3) the elevation of tau, phosphorylated tau, and neurofilament light chains differentiated between PD and the related disorders, and (4) the elevation of cytokines (IL-1β, IL-6, and TGF-β) was observed in PD. Many other candidate biomarkers were also scrutinized. These findings will aid in the accurate diagnosis of PD and other neurodegenerative disorders and facilitate understanding of the pathogeneses of these conditions. Further studies will be needed to obtain more precise measurements of the biochemical biomarker levels. Any single biomarker cannot lead to a ‘snap-shot’ diagnosis. Combinations of several biomarkers would help with more accurate diagnoses and evaluation of the pathophysiology.

CSF biomarkers would aid in understanding the pathophysiological mechanisms, improve the diagnostic accuracy, and facilitate the future development of novel therapies, including disease-modifying drugs.

Finally, we refer to the limitation of this article. This work is a narrative review but not a systematic review. Therefore, we admit that this article could contain some selection bias although each report was fairly scrutinized as much as possible. The aim of this work is to provide the latest comprehensive review regarding cerebrospinal fluid biomarkers of PD, to grade the reliance of each marker according to meta-analyses, and to focus on each marker or marker category with respect to future research directions. We agree that the systematic review method is a more reliable scientific approach. However, referring to all reports offsets the conciseness of this article, and even dopamine and the metabolites, which are generally regarded as central issues in the pathophysiology of PD, have no meta-analysis yet. Hence, we did not adopt the systematic review approach in this article.

## Figures and Tables

**Table 1:** Candidate cerebrospinal fluid biomarkers for distinguishing Parkinson’s disease from controls.

Biomarker	Putative Function/Role/Interpretation	PD vs. Control	Meta-Analysis	References
A. neurotransmitters and neuromodulators				
anandamide	fatty acid neurotransmitter, endocannabinoid	↑		[6]
DA, DOPAC	dopamine and the metabolites	↓		[7]
DHPG, NE	norepinephrine and the metabolites	↓		[7]
Neuromodulin (GAP43)	presynaptic terminal component	↓		[8]
3-hydroxykynurenine	tryptophan metabolite, excitotoxin	↑		[9]
B. oxidative stress markers				
advanced oxidized protein products (self-oxidized)	Protein and amine halogenation	↓		[10]
ceruloplasmin ferroxidase activity	copper-dependent oxidase activity	↓		[11]
oxidized Q10	ubiquinone	↑		[12]
Cu/Zn-superoxide dismutase	associated with ALS1	↓		[11]
copper	metal	→	✓	[13]
zinc	metal	→	✓	[13,14]
manganese	metal	→	✓	[13]
DJ-1	redox-sensitive chaperone, PARK7	↑↓?		[15,16,17]
glutathione S-transferase Pi	glutathione-associated detoxification	↓		[18]
glutathione (oxidized)	antioxidant	↓		[9]
hydroxy radical (OH)	reactive oxygen species	↑		[19]
lipid peroxidation	lipid redox	↑		[11]
nitrites, nitrates	NO metabolites	↑		[11]
silicic acid (si)	silicon compound	↓		[20]
xanthine	purine metabolite	↑		[21]
3-nitrotyrosine products	tyrosine nitration	↑		[22]
8-hydroxyguanosine (8-OHG)	RNA stress marker	↑		[23]
8-hydroxy-2’-deoxyguanosine (8-OHdG)	DNA stress marker	→	✓	[13]
urate or uric acid	purine metabolite, antioxidant	↓		[24]
C. inflammatory and immunological markers				
β2-microglobulin	detected by multiplex proteomics assays	↑		[25]
Immunoglobulin G (IgG) ratio (CSF/serum)	blood–brain barrier permeability	↑		[26]
cytokines (IL-1β, IL-6 and TGF-β)	identified by meta-analysis	↑	✓	[27]
interferon (IFN)-γ	cytotoxic neuroinflammatory factor	↓		[19]
prostaglandin E2	prostaglandin	→		[19]
soluble CD (cluster of differentiation) 14	macrophage marker	→		[28]
tumor necrosis factor (TNF)-α	cytotoxic neuroinflammatory factor	↓		[19]
differentially sialylated isoforms of Serpin A1	serine protease inhibitor	→		[29]
D. Growth factors				
brain derived neurotrophic factor (BDNF)	detected by multiplex proteomics assays	↓		[25]
progranulin	associated with frontotemporal dementia	→		[30]
E. proteins involved in PD pathology				
α-synuclein	Lewy body component	↓	✓	[31,32,33,34]
α-synuclein oligomer, phosphorylated α-synuclein	α-synuclein subspecies	↑	✓	[34]
amyloid β42	Alzheimer’s pathology-related	↓	✓	[35]
total tau, phosphrylated tau	Alzheimer’s pathology-related	↑	✓	[35]
neurosin	alpha-synuclein cleaving enzyme	↓→?		[36,37]
glial fibrillary acidic protein (GFAP)	glial damage	→		[38]
clusterin	clearance of cellular debris and apoptosis	↑		[18]
neurofilamant light chain	neuronal damage	→		[39]
neurofilament heavy chain	neuronal damage	→		[40]
YKL-40 (CHI3L1)	glial marker	↓		[28]
soluble neuron-glial antigen 2 (NG2) proteoglycan	proliferation/migration/differentiation of pericytes etc.	→		[37]
ubiquitin	ubiquitin-proteasome system	→		[41]
UCHL-1	PARK5, deubiquitinating enzyme	↓		[42]
apolipoprotein A1	detected by multiplex proteomics assays	↓→?		[18,25]
apolipoprotein A2	detected by multiplex proteomics assays	↓		[25]
apolipoprotein epsilon	risk factor of Alzheimer’s disease	↑→↓?		[18,25,43]
transthyretin	post-mortem 2D-DIGE assays	↑		[18]
glycan isoforms of transferrin (serum-type/brain-type)	derived from choroid plexus	↑		[44]
neuron-specific enolase	neuronal damage	→		[45]
myelin basic protein	myelin damage	→		[45]
glucocerebrosidase activity	lysosomal enzyme	↓		[46]
F. others				
albumin ratio (CSF/serum)	blood–brain barrier permeability	↑		[6]
corticosterone	post-mortem analysis	↓		[9]
creatinine	GC-TOFMS-based metabolomics and immunoassays	↓		[47]
fibrinogen	post-mortem 2D-DIGE assays	↓		[18]
haptoglobin	detected by multiplex proteomics assays	→		[25]
insulin	glucose regulator	→		[48]
vitamin-D binding protein	detected by multiplex proteomics assays	↑		[25]
xylitol	GC-TOFMS-based metabolomics and immunoassays	↓		[47]
3-hydroxyisovaleric acid	GC-TOFMS-based metabolomics and immunoassays	↓		[47]
tryptophan	GC-TOFMS-based metabolomics and immunoassays	↓		[47]
microRNAs	posttranscriptional regulators	→	✓	[49,50]
exosomes	release and transfer of multiple molecules among cells			[51,52]
miR-1	“dopaminergic synapse” pathway	↓		[51,52]
miR-19b-3p	“dopaminergic synapse” pathway	↓		[51,52]
miR-153	“neurotropin signaling” pathway	↑		[51,52]
miR-409-3p	“neurotropin signaling” pathway	↑		[51,52]
miR-10a-5p	“neurotropin signaling” pathway	↑		[51,52]
Let7g-3p	“neurotropin signaling” pathway	↑		[51,52]
Prolyl oligopeptidase (prolylendopeptidase)	promotion of α-synuclein oligomerization	↓		[53]

↑ = increase, ↓ = decrease, → = no change. ? means inconclusive or conflicting results. 2D-DIGE, 2-dimensional difference gel electrophoresis; 5-HIAA, 5-hydroxyindole acetic acid; ALS1, amyotrophic lateral sclerosis type 1; DA, dopamine; DHPG, dihydroxyphenylglycine; DOPAC, 3,4-dihydroxy-phenylacetic acid; GC-TOFMS, Gas Chromatography-Time-of Flight Mass Spectrometry; HVA, homovanillic acid; IL, interleukin; NE, norepinephrine; PARK5, familial Parkinson’s disease type 5; PARK7, familial Parkinson’s disease type 7; PD, Parkinson’s disease; TGF, Transforming growth factor; UCHL-1, Ubiquitin carboxyl-terminal hydrolase isozyme L1.

**Table 2:** Candidate cerebrospinal fluid biomarkers for distinguishing Parkinson’s disease.

Biomarker	PD vs. Non-PD	Meta-Analysis	References
A. neurotransmitters and neuromodulators			
DA, DOPAC	→		[7]
DHPG, NA	↑(vs. PAF)		[7]
5-HIAA	↑(vs. MSA)		[45]
Neuromodulin (GAP43)	↓(vs. AD)		[8]
orexin	↑(vs. CBD), ↓(vs. PSP)		[54]
B. oxidative stress markers			
ceruloplasmin ferroxidase activity	→		[11]
Cu/Zn-superoxide dismutase	→		[11]
copper	→	✓	[13]
zinc	→	✓	[13,14]
DJ-1	↓(vs. AD, MSA)		[15,16,17]
lipid peroxidation	→		[11]
nitrites, nitrates	→		[11]
8-hydroxyguanosine (8-OHG)	↓(vs. MSA)		[55]
C. inflammatory and immunological markers			
β2-microglobulin	↓(vs. PSP)		[25]
soluble CD (cluster of differentiation) 14	→		[28]
Flt3 (Fms-related tyrosine kinase 3) ligand	↑(vs. MSA)		[56]
D. Growth factors			
brain derived neurotrophic factor (BDNF)	→		[25]
E. proteins involved in PD pathology			
α-synuclein	↓(vs. AD)	✓	[31,32,33,34]
neurosin	→		[36,37]
glial fibrillary acidic protein (GFAP)	→		[38]
neurofilament light chain	↓(vs. MSA, AD)	✓	[57,58,59,60]
neurofilament heavy chain	↓(vs. MSA, PSP)		[40]
YKL-40 (CHI3L1)	↓(vs. PSP, CBD, MSA)		[28]
soluble NG2 proteoglycan	→		[37]
ubiquitin	↓(vs. PSP)		[41]
UCHL-1	↓(vs. MSA, PSP, CBD)		[42]
apolipoprotein A1	→		[25]
apolipoprotein A2	→		[25]
apolipoprotein ε	↓(vs. AD)		[18,25,43]
neuron-specific enolase	↑(vs. MSA)		[45]
myelin basic protein	↑(vs. MSA)		[45]
F. others			
haptoglobin	→		[25]
vitamin-D binding protein	→		[25]

↑ = increase, ↓ = decrease, → = no change. 5-HIAA, 5-hydroxyindole acetic acid; AD, Alzheimer’s disease; CBD, corticobasal degeneration; DA, dopamine; DHPG, dihydroxyphenylglycine; DOPAC, 3,4-dihydroxy-phenylacetic acid; MSA, multiple system atrophy; NE, norepinephrine; PAF, pure autonomic failure; PD, Parkinson’s disease; PSP, progressive supranuclear palsy; UCHL-1, Ubiquitin carboxyl-terminal hydrolase isozyme L1.

**Table 3:** Candidate cerebrospinal fluid biomarkers for representing disease severity or cognitive abilities.

Biomarker	Cognition and Severity	Meta-Analysis	References
A. neurotransmitters and neuromodulators			
DHPG	↓(with orthostatic hypotension)		[7]
B. oxidative stress markers			
advanced oxidized protein products (self-oxidized)	anti-halogenative capacity↓(HY1-2)		[10]
ceruloplasmin ferroxidase activity	positive correlation with onset time		[11]
oxidized Q10	negative correlation with duration		[12]
copper	correlation with disease duration		[11]
zinc	→	✓	[13,14]
DJ-1	↑ in HY1-2 vs. HY3-4		[15]
lipid peroxidation	positive correlation with onset		[11]
3-nitrotyrosine products	correlated with HY		[22]
8-hydroxyguanosine (8-OHG)	negative correlation with duration		[55]
C. inflammatory and immunological markers			
β2-microglobulin	→		[25]
Immunoglobulin G (IgG) ratio (CSF/serum)	correlated with HY		[6]
cytokines (IL-1β, IL-6)	↑ in cognitive impairment		[19]
differentially sialylated isoforms of Serpin A1	↑(in PDD)		[29]
D. Growth factors			
brain derived neurotrophic factor (BDNF)	→		[25]
E. proteins involved in PD pathology			
amyloid β42	↓(in cognitive impairment)	✓	[35]
total tau, phosphorylated tau	↑(in PDD)	✓	[35]
neurofilament light chain	correlated with HY		[39]
UCHL-1	correlated with HY		[42]
apolipoprotein A1	→		[61]
apolipoprotein A2	→		[61]
apolipoprotein ε	→		[61]
heart fatty acid-binding protein	↑		[62]
glucocerebrosidase activity	positive correlation with HY		[46]
neprilysin activity	↓(in PDD)		[63]
F. others			
vitamin-D binding protein	→(in PDD)		[61]

↑ = increase, ↓ = decrease, → = no change. DHPG, dihydroxyphenylglycine; HY, Hoehn-Yahr stage; IL, interleukin; PD, Parkinson’s disease; PDD, Parkinson’s disease with dementia; UCHL-1, Ubiquitin carboxyl-terminal hydrolase isozyme L1.

**Table 4:** Summary of the values of sensitivity, specificity, or the effect size of each marker as reported by the meta-analysis studies.

Biomarker	Marker Change	Sensitivity, Specificity or Effect Size	References
IL-6	↑(PD vs. Control)	Hedge’s g = 0.468 (95% CI 0.049–0.887, *p* = 0.031)	[27]
IL-1β	↑(PD vs. Control)	Hedge’s g = 0.370 (95% CI 0.033–0.707, *p* = 0.031)	
TGF-β	↑(PD vs. Control)	Hedges’ g = 0.472 (95% CI 0.147–0.798, *p* = 0.004)	
α-synuclein	↓(PD vs. Control)	SMD −0.67 (95% CI −0.83 to −0.50, *p* = 0.00001)	[31]
	↓(PD vs. Control)	Sensitivity 0.88 (95% CI 0.84–0.91)	[32]
		Specificity 0.40 (95% CI 0.35–0.45)	
	↓(PD vs. Control)	Sensitivity 0.72 (95% CI 0.60–0.81)	[34]
		Specificity 0.65 (95% CI 0.51–0.77)	
α-synuclein	↓(vs. PSP)	SMD −0.38 (95% CI −0.61 to −0.15, *p* = 0.001)	[31]
	↓(vs. AD)	WMD −0.18 (95% CI −0.26 to −0.10, *p* < 0.0001)	[32]
	↓(vs. AD)	SMD 0.87 (95% CI 0.15–1.58, *p* < 0.05) (* AD vs. PD)	[33]
α-synuclein oligomer	↑(PD vs. Control)	Sensitivity 0.71 (95% CI 0.49–0.86)	[34]
		Specificity 0.64 (95% CI 0.44–0.80)	
phosphorylated α-synuclein	↑(PD vs. Control)	SMD 0.86 (95% CI 0.54-1.18, *p* < 0.001)	[34]
amyloid β42	↓(PDCI vs. PDNC)	SMD −0.44 (95% CI −0.61 to −0.26, *p* < 0.00001)	[35]
total tau	↑(PDCI vs. PDNC)	SMD 0.21 (95% CI 0.06–0.35, *p* = 0.006)	[35]
phosphorylated tau	↑(PDCI vs. PDNC)	SMD 0.36 (95% CI 0.02–0.69, *p* = 0.04)	
neurofilament light chain	↓(vs. MSA)	SMD 1.60 (95% CI 1.22–1.98, *p* < 0.0001)	[57]
	↓(vs. PSP)	SMD 2.04 (95% CI 1.69–2.40, *p* < 0.0001)	[57]
	↓(vs. MSA)	SMD 1.56 (95% CI 1.12–2.00, *p* < 0.00001)	[58]
	↓(vs. APD)	Sensitivity 82% (95% CI 68–91%)	[59]
	↓(vs. APD)	Specificity 85% (95% CI 79–89%)	

↑ = increase, ↓ = decrease, → = no change. AD, Alzheimer’s disease; APD, Atypical parkinsonian disorders; CI, confidence interval; IL, interleukin; MSA, multiple system atrophy; PD, Parkinson’s disease; PDCI, Parkinson’s disease with cognitive impairment; PDNC, Parkinson’s disease with normal cognition; PSP, progressive supranuclear palsy; SMD, standardized mean difference; TGF, transforming growth factor; WSD, weighted mean difference. * Please note this SMD value represents comparison of AD to PD as described in the reference [33].
